# Redox Imbalance in T Cell-Mediated Skin Diseases

**DOI:** 10.1155/2010/861949

**Published:** 2010-08-04

**Authors:** Saveria Pastore, Liudmila Korkina

**Affiliations:** Laboratory of Tissue Engineering and Cutaneous Physiopathology, Istituto Dermopatico dell'Immacolata (IDI IRCCS), Via Monti di Creta 104, 00167 Rome, Italy

## Abstract

The skin is permanently exposed to physical, chemical, and biological aggression by the environment. In addition, acute and chronic inflammatory events taking place in the skin are accompanied by abnormal release of pro-oxidative mediators. In this paper, we will briefly overview the homeostatic systems active in the skin to maintain the redox balance and also to counteract abnormal oxidative stress. We will concentrate on the evidence that a local and/or systemic redox dysregulation accompanies the chronic inflammatory disorder events associated to psoriasis, contact dermatitis, and atopic dermatitis. We will also discuss the fact that several well-established treatments for the therapy of chronic inflammatory skin disorders are based on the application of strong physical or chemical oxidants onto the skin, indicating that, in selected conditions, a further increase of the oxidative imbalance may lead to a beneficial outcome.

## 1. Introduction

Epithelial cells are the outermost viable components of the skin and mucous membranes, and are the fundamental generators of highly specialized physicochemical barriers, that is, organized tissues that exist primarily to define and separate distinct compartments of the body with the aim to protect the host in its entirety and preserve single organ functions homeostatically. These protective functions are obviously maximal in the skin, the conjunctiva and the respiratory mucosa, since they define boundaries with direct contact with the environment. Normally, environmental microorganisms, toxins, and allergens are efficiently blocked from entry by the defence wall organized by layers of epithelial cells. In the viable portion of the epidermis, lipid plasma membranes, and highly specific membrane transport systems pose a formidable obstacle to transepithelial passages of most undesired molecules. In addition, intercellular junctional barrier connections (or tight junctions) create a continuum with the epithelial cell cytoskeleton, and are conserved even when epithelial cells divide or apoptose [1]. However, much of the physicochemical barrier function of human epidermis against the environment is provided by the cornified cell envelope (CE), a unique, highly lipophilic two-compartment system of corneocytes embedded in a lipid-enriched intercellular matrix [2]. Moving upward from the deepest layers of the epidermis, keratinocytes progressively differentiate and eventually become anucleated corneocytes, formed of cross-linked keratins enclosed within an insoluble matrix of proteins and surrounded by a ceramide-rich lipid envelope. Both protein and lipid components of CE are essential for an optimal barrier function, as demonstrated by genetic defects underlying several human diseases and a number of mouse models [3, 4]. 

## 2. Mechanisms of Redox Balance in the Skin

**Figure 1:**
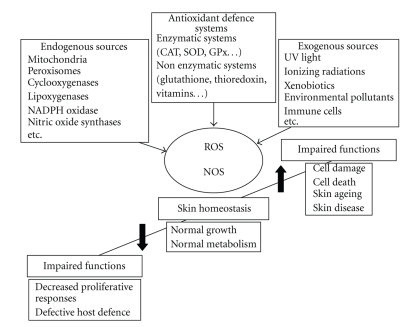
Sources and responses to reactive oxygen and nitrogen species (ROS and RNS) in the skin. These highly reactive entities are generated as a result of normal intracellular metabolism in mitochondria and peroxisomes, and by a variety of cytosolic enzyme systems. The skin is peculiarly rich of enzymatic and nonenzymatic systems for the regulation of overall ROS and RNS levels and hence for the maintenance of physiological homeostasis, due to its direct contact with strong pro-oxidative physical and chemical insults from the environment. These include UV and ionizing radiations, and a variety of pollutants. During chronic inflammatory events, the persistent release of potent cytokines by infiltrating leukocytes contribute to perturb the redox balance, essentially through the upregulated expression of numerous enzymes involved in the regulation of this balance. Lower-than-normal levels of ROS and RNS leads to impaired cell proliferation and reduced host defence. Also an increase in their levels is detrimental for the skin, leading to damage to cell components and eventually acceleration of ageing and age-related diseases.

In the healthy skin, practically all types of skin cells produce reactive oxygen (ROS) and reactive nitrogen (RNS) species [5]. For example, both melanocytes and keratinocytes produce hydrogen peroxide (H_2_O_2_) and superoxide radicals in the reaction of pheomelanin with UV light [6]. All cells also produce small amounts of superoxide anion as by-product of electron transfer in the mitochondrial respiratory chain. By reacting with lipid molecules directly, or affecting redox-sensitive lipid-metabolizing enzymes (phospholipases, lipoxygenases, and cyclooxygenases), ROS induce production of reactive lipid species (lipid radicals, peroxides, hydroperoxides, aldehydes, etc.). Although dismutation of superoxide anion probably accounts for much of the H_2_O_2_ produced by eukaryotic cells, it can also be formed by direct two-electron reduction of oxygen, a mechanism shared by a number of flavoprotein oxidases [7]. Importantly, nitric oxide (NO) and peroxynitrite are major RNS in biological systems. In the skin, NO is produced by two constitutive nitric oxide synthase (cNOS) isoforms, identified as endothelial (eNOS) and neuronal (nNOS), and one inducible (iNOS) isoform. Both fibroblasts and keratinocytes constitutively express eNOS. NO easily reacts with oxidative species, including ROS, transition metals and thiols to yield various RNS. In particular, NO reacts with superoxide anion at near diffusion-limited rates, leading to the extremely rapid production of the peroxynitrite ion. When generated at high concentrations, peroxynitrite can diffuse and undergo transformation into other powerful oxidants, including the hydroxyl radical (OH·) and other RNS (NO_2_, NO_2_^+^) [8]. It is important to emphasize that low levels of highly reactive ROS and RNS are indispensable effectors in the homeostatic pathways leading to cell proliferation, differentiation, senescence, and death, and a vast body of evidence confirms that this is true also for the distinct cell populations of the human skin, as extensively commented elsewhere [5]. Due to its direct contact with environmental physicochemical challenges, the skin is peculiarly rich of effective antioxidant systems (Figure 1). In the viable layers of the epidermis, lipid-soluble antioxidants, mainly alphatocopherol, and antioxidant enzymes like catalase (CAT), superoxide dismutases (SOD), glutathione peroxidases (GPx), and peroxiredoxins are abundantly expressed [9]. The extracellular space of skin epidermis and dermis, contains large amounts of water-soluble antioxidants such as ascorbic acid, uric acid, and glutathione. In addition to effectively counteract insults coming from the environment, the CE of normal human skin contains high levels of water and lipid-soluble antioxidants such as glutathione, thioredoxin, vitamin C, uric acid, *α*-tocopherol, squalene, and coenzyme Q10, distributed in a gradient with the highest concentration on the deepest CE layers [10]. As part of an adaptive response, abnormal levels of ROS also induce the expression of ROS-detoxifying enzymes. In order to protect the structural and functional integrity of the skin, a wide spectrum of phase I enzymes, active both in oxidation and reduction, and phase II enzymes, active in conjugation reactions, can be found upregulated in the skin, or can be rapidly induced in response to different physical and chemical agents. The transcriptional response to these agents is typically mediated by the *cis*-acting antioxidant response element (ARE), found in the promoter of the encoding genes for diverse products such as several glutathione S-transferases, metalloproteinases, NADPH:quinone oxidoreductase 1, UDP glucuronotransferase, *γ*-glutamate cysteine ligase, heme oxygenase 1, and peroxiredoxin VI. The major ARE-binding transcription factor is nuclear factor-erythroid 2-related factor 2 (Nrf2), which, through heteromeric interaction with the small Maf proteins, binds the ARE and initiates the *de novo* expression of detoxifying enzymes [11]. In the skin, although several Nrf2-dependent ROS-detoxifying enzymes are found upregulated in wound healing, abrogation of Nrf2 expression in transgenic mice is substantially irrelevant for the healing process, whereas it is essential for the effective detoxification of chemical carcinogens [12]. These observations emphasize the special chemopreventive role of Nrf2-controlled genes in the skin. The transcriptional activation of Nrf2-dependent genes can be induced by various chemicals, including redox-active compounds such as quinones, isothiocyanates, peroxides, mercaptans, transition metals, trivalent arsenicals, and also by chemopreventive antioxidants such as dietary polyphenols [11]. By contrast, chemopreventive polyphenols may oppose inflammation and cancer by enhancing cellular antioxidant and detoxifying enzymes *via* activation of Nrf2. They also suppress the induction of proinflammatory and growth-promoting genes by downregulating the activation of the crucial transcription factors Nuclear Factor *κ*B (NF*κ*B) and activator protein 1 (AP-1) [13]. In response to a variety of oxidant reactants, the skin upregulates transactivating AP-1 components such as Fos and Jun, whereas it downregulates its negative, anti-inflammatory components Fra-1 and Fra-2. The shift of redox balance towards oxidative conditions facilitates phosphorylation and activation of c-Jun N-terminal kinase (JNK), a kinase that promotes Jun translocation into the nucleus. As a result, transcription of AP-1-dependent genes takes place and, if this process persists, cells will eventually die by apoptosis. Ascorbic acid is an antiapoptotic antioxidant, which provides redox-dependent inhibition of JNK and activation of Fra-1 and Fra-2. A detailed analysis of the redox modulation of the signal transduction pathways and transcription factors involved in the inflammatory response of the skin has been recently published [14]. 

## 3. Redox Imbalance in T Cell-Mediated Skin Inflammation

T cell-mediated diseases of the skin are chronic disorders with a high-social impact and an increasing prevalence in the western countries. These diseases include psoriasis, contact dermatitis, and atopic dermatitis. In the last decades, relevant advances have been achieved in the understanding of the cellular and molecular mechanisms underlying these disorders [15]. Similar to the inflammatory events taking place in other organs, also chronic skin inflammation associates with overproduction of ROS, RNS, and high levels of products of lipid peroxidation. Here, we will briefly review recent acquisitions on the pathogenesis of T cell-mediated skin disorders, and the evidence of disease-specific redox imbalance.

### 3.1. Pathogenesis of Psoriasis

Psoriasis is characterized by an intrinsically dysregulated interrelation between keratinocytes and cells of both the innate and acquired immune response [16]. As a result, psoriatic keratinocytes aberrantly proliferate and become a reservoir of inflammatory mediators that, directly and indirectly, induce the psoriatic phenotype. In the last years, a number of studies have highlighted the central involvement of dermal dendritic cells (DCs) in the pathogenesis of psoriasis. In particular, dermal myeloid DCs are increased in psoriatic lesions and induce T cell proliferation and the massive production of the type 1 cytokine interferon (IFN)-  *γ* [17]. A specialized subgroup of these cells is named tumor necrosis factor (TNF)-*α* and iNOS-producing DC (TIP-DC), due to their high expression of these inflammation markers [18]. The evidence that targeted immunotherapy reduces the quantity of these cells in psoriatic patients supports the concept that these cells are associated to the disease and have a pathogenetic role [18, 19]. Importantly, TIP-DC can also produce IL-20 and IL-23, and stimulate the differentiation and activation of IL-17-producing helper T cells (Th17) [20]. Th17 cell type is specialized in the immunosurveillance of the epithelium, and also secretes high levels of IL-22, a key cytokine linking adaptive immune effectors and epithelial dysregulation in psoriasis. Indeed, IL-22, rather than IL-17A, from which Th17 cells are named, is emerging as the prototypic Th17 cytokine [21], by inducing proliferation of keratinocytes and synergizing with IL-17 in the enhanced expression of antimicrobial peptides and chemokines by skin keratinocytes [21, 22]. Hence, IL-22 could be centrally involved in the pathogenesis of psoriasis, a disease characterized by very high levels of numerous chemokines and antimicrobial peptides [16]. A first functional role of Th17 cells in psoriasis was suggested by reduction of their number during successful anti-TNF treatment [23, 24]. In addition, a population of plasmacytoid DC (pDC) is now thought to be crucially implicated in the early phases of the psoriatic plaque formation. These cells release IFN-*α* in the skin, and this event is currently believed to trigger the expansion of autoimmune T cells. In humans, infiltration of pDC and expression of IFN-*α* can be detected early and transiently during the development of the psoriatic plaque whereas its effect persists in time until lesion chronicization [25]. In contrast, pDC are almost absent in chronic psoriatic lesions. Also the expression of chemerin, a chemotactic factor active specifically on pDC recruitment through its binding to the Chemerin R23 receptor on their surface [26], can be observed only in association with early pDC infiltration in psoriatic skin [25].

### 3.2. Evidence of Oxidative Stress in Psoriasis

There are many systemic and lesion-restricted signs of severe oxidative stress in the patients with active psoriasis [27]. In the plasma and red blood cells of patients with active psoriasis, increased levels of malonyl dialdehyde (MDA) were interpreted as the fingerprint of the exhaustion of natural enzymatic and nonenzymatic antioxidant defenses and consequently the prevalence of deleterious peroxidative processes in the cell membranes and plasma lipids of circulating cells [28, 29]. In another study, erythrocytes from psoriatic patients presented a statistically significant decrease in erythrocyte SOD and GPx [30]. In this report, exaggerated levels of MDA were measured in the lesional tissues but not in the serum of psoriatic patients. Other authors described a decreased antioxidant potential of the plasma, along with higher-than-normal expression of SOD, and elevated MDA levels but no correlation between these parameters and the disease severity [31]. Taken together, these results support the notion that an imbalance in the oxidant-antioxidant system can be observed in psoriatic patients [32]. In the skin lesion, the massive infiltration of the various leukocyte populations in an activated state certainly leads to local release of a number of pro-oxidative species, in their turn implicated in the proinflammatory activation of the resident cells of the skin, in particular, keratinocytes and fibroblasts [14]. 

Interestingly, several proinflammatory mediators remarkably upregulated in the psoriatic lesions, in particular TNF-*α*, IFN-*γ*, and IL-8, are strong inducers of iNOS expression and NO release from the epidermal keratinocytes [33, 34]. In addition, psoriatic lesions are peculiarly infiltrated by TIP-DC, characterized by abundant levels of iNOS, as previously described in [18, 19]. Being a potent regulator of keratinocyte growth and differentiation, NO has been recently considered a key player in the pathogenesis of psoriasis. Indeed, a large body of work suggests that NO is proinflammatory in the skin. Application of an NO-releasing cream on the skin of healthy subjects elicits an inflammatory response, and a loss of Langerhans cells that was correlated to upregulated iNOS activity during the UVB-induced erythematous response [35, 36]. However, other investigators found that application of an NO-releasing ointment on active psoriatic lesions leads to disease regression, possibly due to prominent NO-dependent downregulation of chemokine expression and hence to inhibition of T cell and macrophage attraction into the skin [37]. This last report emphasizes a possible beneficial effect of NO on this chronic disorder, and suggests that the actual levels of NO are lower than normal in psoriatic lesions. Accordingly, the evidence that arginase 1 is aberrantly overexpressed in the lesional skin of psoriasis patients suggests the possibility that arginine, which is the substrate for NO generation by iNOS, might not be adequately available for iNOS activity [38]. Also an abnormal generation of the superoxide anion could limit NO concentration in the psoriatic lesion, since this species may rapidly react with NO and generate peroxynitrite, a cytotoxic agent that nitrosylates thiolic groups and also leads to DNA break [39]. Of relevance, exogenous NO was shown to exhibit a biphasic action on HaCaT keratinocytes, providing a proliferative signal at lower concentrations (below 100 micromolar), and inducing cell cycle arrest at higher doses [40]. 

In spite of the evidence that an oxidative stress is systemically and locally present during this disease, a variety of effective treatments currently used for the therapy of psoriasis rely on a boost of the oxidative stress itself. For example, psoralen-UVA (PUVA) combined therapy is widely used in the treatment of psoriasis, and it leads to massive generation of singlet oxygen in the skin [41]. Solar irradiation is also known to improve the clinical conditions in a considerable proportion of patients with psoriasis [16]. In the whole human skin in vivo, the sun light induces production of IL-6 accompanied by increased levels of 8-isoprostane, a marker of oxidative damage to skin lipids [42]. Also broad-band UVB phototherapy was successfully introduced into therapeutic protocols for psoriasis. UVB phototherapy leads to a sharp increase in the levels of thiobarbituric acid (TBA) products and nitrite/nitrate concentration in the plasma of patients subjected to a 1 to 2-month-long treatments [43]. In patients affected by severe forms of psoriasis, systemic treatments include methotrexate, an inducer of elevated nitrite/nitrate levels in the serum [44]. Fumaric acid esters are also used for the systemic therapy of psoriasis with good clinical efficacy. Elevation of superoxide anion production by circulating blood monocytes in patients treated with this drug was documented [45]. Finally, anthralin is a well-established topical treatment for psoriasis. Its positive clinical effect seems to associate to hydrogen peroxide-induced activation of EGF receptor in keratinocytes [46]. Pro- and antioxidant properties of anthralin and some of its derivatives have been characterized [47]. In their whole, these observations suggest that a further increase of the local levels of oxidative imbalance can play a beneficial, anti-inflammatory effect in the skin of psoriatic patients, possibly due to activation of antiproliferative, proapoptotic pathways in both resident and infiltrating cell populations.

### 3.3. Pathogenesis of Allergic Contact Dermatitis

Allergic contact dermatitis (ACD) is a common inflammatory skin disease, induced by repeated contact with low molecular weight, highly reactive chemicals called haptens [48]. This disease affects about 5% of men and 11% of women in industrialized countries and has a chronic evolution. While the irritant contact dermatitis (ICD) is a nonspecific inflammatory dermatosis mainly due to severe damage to the epidermal barrier and/or direct toxicity of the xenobiotics or chemicals on the skin cells, ACD is a delayed-type hypersensitivity response, with skin damage eventually due to activation of the acquired immunity and infiltration of the tissues by a rich population of lymphocytes including hapten-specific T cells. Indeed, haptens irreversibly change the chemical nature of endogenous proteins, and hence trigger an antigen-specific response in the course of a second re-exposure. The cellular mechanism behind this adaptive response has been deeply investigated in the last decades [15]. Hapten interaction with extracellular or membrane-bound proteins is followed by internalization into skin antigen presenting cells, mostly Langerhans cells and dermal DCs, and processed into major histocompatibility complex (MHC) class II compartments to generate appropriate hapten-peptide complexes fitting in the groove of MHC molecules. Skin DCs reside in the skin in an immature state, characterized by their high capacity to capture antigens penetrated through the CE, but very low-antigen presenting capacity [49, 50]. Both Langerhans cells and dermal DCs are susceptible to environmental stimuli such as irritants, toxic stimuli, and bacterial and viral products, which promote their migration to regional lymph nodes and the acquisition of antigen presenting capacity for naïve T-cell priming and differentiation. Cytokines released by skin resident cells, in particular IL-1, TNF-*α*, and IL-18, critically regulate both DC migration and functional maturation [51]. Once migrated into the paracortical zone of draining lymph nodes, skin DCs prime hapten-specific naïve T lymphocytes, which differentiate into memory/effector T cells and acquire defined migratory behavior towards the skin, so that they will specifically migrate into the skin upon a further contact with the same hapten. Similar to what can be found in the psoriatic lesion, high levels of TNF-*α* and IFN-*γ* are released by activated leukocytes also in ACD, and contribute to stimulate a strong proinflammatory program in the resident cells of the skin.

### 3.4. Evidence of Oxidative Stress in ACD

Oxidative stress is regarded as widely implicated in the molecular mechanisms leading to both ICD and ACD [52]. Examples of irritants or antigens with overt oxidizing behavior are numerous, and include hydroperoxides and peroxides, metal salts such as nickel (II) or chromium (VI), quinines, and primary amines [53]. All these substances may determine oxidative stress in the cell and dysregulate its molecular mechanisms, or may themselves require redox metabolism to become antigenic. The proinflammatory activity of ROS-generating chemicals in the skin is exemplified by the induction of ICD through intradermal injection of hydrogen peroxide-producing enzymes including glucose oxidase, a reaction that can be neutralized by simultaneous administration of CAT or SOD [54]. Systemic or topical treatment with N-acetylcysteine prior to epicutaneous application of the strong sensitizer and frankly oxidant compound 2,4,6-trinitro-1-chlorobenzene, reduces all the inflammation-correlated parameters, including expression of proinflammatory cytokines, edema, and extent of leukocyte infiltration [55]. The group of strong sensitizers with a dinitrohalobenzene structure, namely, 2,4-dinitro-1-fluorobenze and 2,4-dinitro-1-chlorobenzene, were shown to irreversibly inhibit mammalian thioredoxin reductase [56], hence eliminating thioredoxin contribution to the maintenance of intracellular redox balance. In the positive patch test for ACD to nickel (II), an abnormal elevation of the oxidized/reduced glutathione ratio and an increase in the levels of iron can be detected in the skin lesion [57]. In contrast, induction of ACD to polyaromatic hydrocarbons requires the biosynthesis of a reactive oxidative intermediate by cytochrome P450-dependent enzymes [58]. Also paraphenylenediamine, a very diffuse agent of ACD in the industrialized world, requires a cytochrome P450-dependent enzyme to become reactive and elicit the disease [59]. Finally, the depletion of the enzymatic and/or nonenzymatic antioxidant defenses precipitates the cells in a condition of oxidative stress, which represents a robust trigger for the activation of a proinflammatory response. In particular, activation of the redox-sensitive NF*κ*B pathway leads to the de novo expression of a plethora of cytokines and chemokines by resident cells [53, 60] and eventually to tissue damage, due to massive leukocyte infiltration. 

An increase of iNOS was found immunohistochemically both in ICD and ACD [61], and hence NO can reasonably play a role in the distinct processes going on in contact with dermatitis, including enhanced proliferation of epidermal cells, neutrophil accumulation and local vasodilatation [62]. Indeed, iNOS blockade by aminoguanidine prior to immune challenge was shown to reduce the inflammatory response to the sensitizers picryl chloride and 2,4-dinitro-1-fluorobenze [63, 64]. In humans, topical application of an NO-releasing cream provides a sufficient stimulus to induce ICD, as previously discussed in [36], although an independent investigation suggests that NO exerts a more complex regulation of the inflammatory skin reactions, with potentiation of immune-specific cell-mediated mechanisms, but limited role during ICD [65].

### 3.5. Pathogenesis of Atopic Dermatitis

Atopic dermatitis (AD) is a chronic inflammatory disease that results from complex interactions between genetic and environmental factors [66]. A defect of total lipids of the CE, including sterol esters and phospholipids as well as an increase in free fatty acids and sterols compared to normal controls, is responsible of the xerotic aspect of the atopic skin, and may determine a higher permeability to allergens and irritants, including environmental toxins. Recent findings have provided evidence that a disturbed protease-antiprotease balance is also implicated in a faulty differentiation process of keratinocytes and accelerated desquamation in this disease [66]. However, the strongest evidence for a primary structural abnormality of CE underlying the pathogenesis of AD derives from the recent link between loss-of-function mutations in the gene encoding filaggrin and AD [67, 68]. Up to 50% of European children with AD reveal single or double allele, or compound mutations in the filaggrin gene on chromosome 1q21. Although 15 distinct mutations have been reported, the 2 most common account for the majority of cases [69], and because of their proximal location on the filaggrin gene, they also predict more severe loss of function. Filaggrin deficiency in the epidermis of AD patients is reasonably implicated in different, not yet clearly defined, aspects of CE malfunction in these subjects, including poor CE hydration and decreased generation of filaggrin acidic metabolites such as transurocanic acid, with consequent uncontrolled activation of serine proteases [70]. Any perturbation of the epidermal permeability barrier represents *per se* an effective mechanism leading to cutaneous inflammation, since numerous cytokines, chemokines, and some of the growth factors released by keratinocytes as autocrine regulators of barrier homeostasis can also favor the development of inflammatory reactions [71]. Of note, keratinocytes of AD patients exhibit propensity to an exaggerated production of distinct cytokines and chemokines; a phenomenon that can be relevant in further promoting and maintaining local inflammation [66]. As a consequence, specific immune responses against a variety of environmental allergens are often present, with a bias towards T helper 2 (Th2) immune responses. Atopic diseases are indeed characterized by IgE hyperresponsiveness to environmental allergens and a peculiar hyperreactivity of the target tissues toward irritative/inflammatory stimuli.

### 3.6. Evidence of Oxidative Stress in AD

Factors predisposing susceptible individuals to atopic diseases include chronic exposure to oxidative toxins, such as tobacco smoke and air pollution [72]. Also the relevance of the psychological stress as a risk factor for the precipitation of the atopic syndrome is widely accepted, although the mechanisms of this immunoregulation are poorly understood [72]. Importantly, there is intriguing experimental evidence that chronic psychological stress augments oxidative tissue damage, as demonstrated by the increase of 8-hydroxy-deoxyguanosine (8-OH-dG), a parameter of oxidative DNA damage, in the plasma of animals undergoing permanent painful stimuli [73]. In children with acute exacerbation of atopic dermatitis, urine concentrations of 8-OH-dG, of acrolein-lysine adducts (markers of lipid peroxidation), and of bilirubin oxidative metabolites (markers of bilirubin oxidative metabolism) were higher than those of control subjects, and returned to normal levels during disease remission. In contrast, nitrite/nitrate levels (markers of endogenous nitric oxide production) were not significantly affected in these patients [74]. Upregulation of urinary 8-OH-dG was reported in adult patients undergoing exacerbation of the disease [75]. These findings suggest that AD patients experience an oxidative stress, and that altered antioxidant defences are possibly involved in the pathophysiology of the acute form [28, 32]. Evidence of enhanced protein and lipid-oxidative damage was found in the CE of atopic patients, as demonstrated by the increase of carbonyl moieties both in lesional and nonlesional skin, along with an elevation in the activity of SOD, an effective scavenger of ROS, possibly as part of a mechanism that tends to buffer the persistence of oxidative stress [76]. Recent animal data support a role for oxidative/antioxidative imbalance in the shift toward a Th2-skewed immune response [77]. Accordingly, a previous report demonstrated that administration of the antioxidant thiol N-acetyl-L-cysteine or glutathione to human T cells in culture downregulated the Th2 polarization, with decrease in the expression of IL-4 and IL-5 and simultaneous skewing towards a Th1-type phenotype [78]. These observations indicate that antioxidant supplementation could represent a good therapeutic approach for the treatment of AD. Also the topical use of a proper antioxidant onto the lesional skin could reasonably help to dampen the aberrant keratinocyte expression of cytokines and chemokines in response to proinflammatory stimuli [79]. However, the potential benefit of topical antioxidant treatments in preventing relapses of the disease or in accelerating its remission, has not been clinically assessed. By contrast, similar to psoriasis, severe AD can benefit of exposure to UV light and treatment with PUVA [80], due to a system of responses that lead to effective local immunosuppression. 

## 4. Conclusions

In this paper, we have briefly overviewed our current knowledge of the systemic and local redox imbalance in chronic skin inflammation. The experimental evidence collected so far suggests that the pattern of redox perturbation is disease-specific, and that several well-established, effective pharmacological treatments display a frank pro-oxidant behaviour. In particular, a number of drugs and physical approaches used in the therapy of psoriasis behave as strong systemic and/or local oxidants. In this case, oxidation-induced damage to the cell cycle machinery of hyperproliferating keratinocytes, and of massive numbers of infiltrating leukocytes might eventually perform a valid immunosuppression that helps reduction and/or remission of the plaques. Of note, UV-based therapy is also effective in the therapy of severe AD. Nonetheless, the anti-inflammatory behaviour of numerous natural polyphenols on a variety of cell populations including skin cells, could be ideally employed to protect the skin from abnormal response to environmental triggers and prolong the disease-free periods [81]. To reach this goal, a further step forward in the investigation of the complex, molecule-specific mechanisms of action of natural antioxidant compounds is necessary, along with double-blind placebo-controlled clinical trials to establish parameters of efficacy and safety of these compounds.

## Abbreviations

ACD: Allergic contact dermatitis;
AD: Atopic dermatitis;
AP-1: Activator protein 1;
ARE: Antioxidant response element;
CE: Cornified envelope;
GPx: Glutathione peroxidase;
ICD: Irritant contact dermatitis;
IFN-*γ*: Interferon *γ*;
JNK: c-Jun N-terminal kinase;
MDA: Malonyl dialdehyde;
NOS: Nitric oxide synthase;
NF-*κ*B: Nuclear factor *κ*B;
Nrf2: Nuclear factor-erythroid 2-related factor 2;
8-OH-dG: 8-hydroxydeoxyguanosine;
RNS: Reactive nitrogen species;
ROS: Reactive oxygen species;
SOD: Superoxide dismutase;
TNF-*α*: Tumor necrosis factor *α*.
